# The Vomiting of Pregnancy

**Published:** 1886-06

**Authors:** John Meredith

**Affiliations:** Wellington, Somerset


### Clinical Records.

---

THE VOMITING OF PREGNANCY. By JOHN MEREDITH, M.D. Edin., Wellington, Somerset.

Severe cases of vomiting in pregnancy—that is, such as justly cause anxiety and alarm as to the life of the patient—are not, as far as my observations go, frequent in the experience of a general practitioner.

The following case seemed to me, at the time I was attending upon it, to possess points of interest different from the usual run: so much so, that I thought it right at the time to make notes of its history and progress.

The case is that of Mrs. F. W., aged 28, married on December 2nd, 1882. Was in domestic service until her marriage; is an active, intelligent person, finding plenty of employment in looking after her house and assisting her husband in his trade, which is that of a dairyman.

She considered herself healthy, only that she had "bilious fits" now and then; but did not vomit when suffering from them, although she felt sick. Her mastication of her food was and had been faulty for a long time, owing to the bad condition of her teeth,—which amply, as I think, accounted for her "bilious fits." There is nothing to note in regard to her family history. Her house is clean and dry. The water supply is not of the best quality; still, it is considered equal to average for such as can be obtained from town pumps.

At the end of a small garden at the back of the dwelling is the wash-house, where Mrs. F. W. passed some of her time each day. It is situated about fifteen yards from the house door. Near this outhouse I found a broken drain connected with some water-closets belonging to houses near. The smell from this drain Mrs. F. W. complained of as being at times disgusting, and made her "quite sick."

She came to see me on the 6th of August, 1884, on account of one of her "bilious fits." I had often attended her before for a similar ailment; but the medicines—a nitro-mur. acid mixture—which I gave her afforded her no relief on that occasion.

On the 10th, four days later, I saw her at her house, and found her suffering from nausea and vomiting. She had brought up some blood the day before. She would not admit the probability of her being in the family-way. I learnt that she had not menstruated since the last couple of days of June, and then it was not much, and that "clotty." The patient said she experienced no particular discomfort until the middle of July, when sickness began, with loss of appetite. And these conditions continued; but she put it all down to biliousness. She attached no significance to the fact that the menses did not appear at the end of July: it was nothing new for her to go now and again a great deal beyond her time, everything coming right afterwards.

However, on the 10th of August I suspected that it was a case of commencing pregnancy, notwithstanding my patient's assurance to the contrary. The appearance of a slight pinkish areola around the nipples rather tended to strengthen my suspicion.

On the 15th I made an examination per vaginam, and found the uterus anteflexed, cervix hard, os tilted backwards, its anterior lip rigid and thicker than the rest, and there was a fulness at the base of the cervix greater than I should have expected to find in an unimpregnated normal uterus.

I saw the patient frequently. I tried every remedy, with the view of stopping the sickness, that I could think of; viz, ipecacuanha in small doses; bicarbonate of soda and hydrocyanic acid; creosote; bismuth; opium, in pills and otherwise; phosphoric acid, and the much-praised new remedy, ingluvin: but all absolutely to no good purpose. The vomiting persisted, and often blood was thrown up. Hot fomentations and poultices over the epigastrium gave some temporary relief. There seemed to be a circumscribed tender spot at the pit of the stomach; but I noticed that this tender spot was not always in the same place, according to the sufferer's description. Anyhow, her condition was becoming alarming and a cause of anxiety.

On the 23rd I tried dilatation of the os uteri by finger pressure; but the state of the part was too rigid for me to make any impression upon it in that way. Next I passed a uterine sound for a short distance into the os, and tried dilatation with its assistance; but the patient became restless, and I had to abandon it, and she assured me at the same time that she did not consider herself in the family-way at all; that consequently her illness did not arise from pregnancy.

Under the circumstances, it was not a matter of surprise that a medical friend who kindly saw the case with me at this time considered it one of gastric ulcer. However, I could not concur in this view, and resolved upon varying my treatment a little. I had the patient fed by means of enemata, at first only partially; but from the 24th of August to the 4th of September, entirely. Still the vomiting persisted, with now and then streaks of fresh blood and bilious green mucus. Small quantities of morphia were introduced with the enemata; but they afforded hardly any relief or rest to the patient, whose condition was getting worse every day.

After some persuasive efforts I got the patient to consent to my trying dilatation of the os uteri again, and on the 3rd of September I introduced an ordinary sponge tent into it and left it there for four hours. The dilatation caused by it was not much; but that night, after its removal, Mrs. F. W. called for some beef tea, which she partook of, and was able to retain and enjoy,—the first and only bit of encouraging improvement observed for over a month. This, unfortunately, was only of short duration; for on the 5th the vomiting and sickness returned as bad as ever, throwing the patient and her friends into a state of despondency: and no wonder!

Thinking that the recurrence of the vomiting might be due to the dilatation of the os uteri not being carried to a sufficient extent, I resolved to introduce another sponge tent. Before doing this I had to overcome a good deal of opposition on the part of the patient, as well as on that of her husband, who had made up their minds that nothing more could be done.

As soon as I obtained something like a tacit consent, I proceeded to introduce the tent. I tried a short one first, but found it difficult to introduce; so I threw it aside and selected one of greater length, which was easily passed into the orifice. This was done in the course of the morning. The sponge tent was left in for nine hours. It caused a certain amount of dilatation, not quite as much as would admit the point of the index finger; still it was a good deal more than was caused in the first instance. The effect upon the patient was good, a matter of unmixed satisfaction to her as well as to those in attendance on her. She rested well that night; swallowed and kept down more than a pint of milk.

There was very little sickness after this. Mrs. F. W. was able to take more and more food every day, and retain it. No pain was experienced after taking food, nor any tenderness on pressure over the region of the stomach. No medicine given.

The case progressed favourably in every way, and the patient was not long before she was fully satisfied that she was pregnant. Her strength returned speedily, enabling her to occupy herself with her usual household duties.

On the 13th of March, 1885, she was delivered of a son, after being in labour 48 hours. The confinement was in every way normal, except perhaps for the length of time. She was of opinion that the child was born fully a fortnight before his proper time. Both mother and child progressed satisfactorily, and both are well and hearty now, more than a year after the event.

*Remarks*, in such cases as this, may not be much to the reader's liking; nevertheless I am tempted to offer one or two which suggested themselves, both during its progress and since.

There is a report of a discussion upon this disorder in the *British Medical Journal* of the 20th December, 1884; and Dr. Wynn Williams mentioned, in the course of his observations, that he was successful in stopping the vomiting by introducing a sound into the os uteri. This action, as I have stated, was not successful in my hands, any more than pressure of the finger against the os externum was,—a plan, I think, recommended by Dr. Copeman long ago.

The slight dilatation caused by the first tent was only momentarily successful, as the altered nervous action induced by it was not sufficient to be sustained, whereas the greater dilatation by a subsequent one enabled the system again to assert its equable and normal innervation.

What the necessary amount of dilatation should be in such cases, I cannot venture to say: probably it varies according to the character of the case and of the patient. An uterine sound could hardly bring about the extent of opening that followed the insertion of the second tent, yet the sound was sufficient in Dr. Wynn Williams' case.

A great deal was said during the discussion referred to about flexion of the uterus; but nothing very clear and decided—at least, nothing as definite as Dr. Graily Hewitt's assertion elsewhere, that "the almost universal cause of the vomiting of pregnancy" is displacement of the uterus.

Applying this to my case, it may fairly be inferred that the tent, in causing the dilatation, brought about a rectifying of the flexed condition, and so removing the cause of the disorder, the good results followed; but, if so, the altered condition must have been very slight, for I was not able to appreciate it by mere digital examination: therefore the remarks cannot stop here.

I have nowhere seen it mentioned that inhalation of sewer air, or exposure to noxious unhealthy environment, might be a potent factor in bringing about the ill complained of; yet I venture to think that that exercised a powerful influence in this case. I have stated that Mrs. F. W. used to suffer often from bilious headache and nausea owing to the irritability of the stomach, brought about by imperfect mastication. There was, in consequence, a condition of disturbed innervation in one of the areas supplied by the pneumogastric nerve—a susceptibility to vomiting had been established. But at the commencement of pregnancy, as well as before, the patient was exposed to an offensive drain smell, which found access to another and a very important surface supplied by another portion of the same nerve, and tended to aggravate the liability to vomiting. Now, it is a matter of common observation, that there are not a few upon whom one of the first effects of sewer air is to produce sickness. Mrs. F. W. was liable to slight bloody discharge from her throat; and when she had a bad cold, in January, 1884, she coughed up a good deal of it from her throat. It all came from the pharynx and nasal passages, and the whiffs of sewer gas which she off and on experienced about her washhouse were, I used to think, the main excitants of this. When pregnancy commenced, and a third portion of the pneumogastric nerve became excited and disordered in its action, it proved like the final straw in the burden, and sufficed to bring about what threatened to be a disastrous breakdown.

Taking all these points into consideration, it seems to me that the flexion, in the marked form in which I discovered it, was more probably the consequence of the vomiting, rather than its cause.

That Mrs. F. W. suffered to some extent from womb displacement—that is, from that ideal normal, not very common, as far as I can learn, in the actual life of ordinary women—goes without being said, since she passed "clotty" discharge at her periods.

